# Physiological Scaffold Remodeling in the Coronary Artery After 30 Months of Bioadaptor Implantation

**DOI:** 10.1016/j.jaccas.2024.103089

**Published:** 2025-03-05

**Authors:** Shiori Kawakami, Akihiko Takahashi, Norimasa Taniguchi, Takeshi Yamada, Tetsuya Hata, Shunsuke Nakajima, Shigeru Saito

**Affiliations:** a Department of Cardiology, Sakurakai Takahashi Hospital, Kobe, Japan; b Graduate School, Kobe Women’s University, Kobe, Japan; c Department of Cardiology, Shonan Kamakura General Hospital, Kamakura, Japan

**Keywords:** Bioadaptor, coronary artery scaffold, coronary artery uncaging, intravascular imaging

## Abstract

The DynamX Bioadaptor (Elixir Medical Corporation) is a novel coronary artery scaffold consisting of 3 helical strands connected by a bioabsorbable polymer. After 6 months, the polymer dissolves, restoring the physiological motion of the coronary artery as the interconnected (caged) structure of the scaffold disengages. Here we report the cases of 2 patients treated with the Bioadaptor who underwent intravascular imaging at the time of the index procedure and during follow-up periods of 30 and 36 months, respectively. The cross-sectional images of the scaffold were analyzed and compared with those obtained during the index procedure. At follow-up, the mean cross-sectional area of the scaffold was increased significantly in both cases, regardless of the degree of intimal hyperplasia. Such unique properties of the Bioadaptor make it a promising alternative to contemporary drug-eluting stents.

## Graphical Abstract

**Figure:**
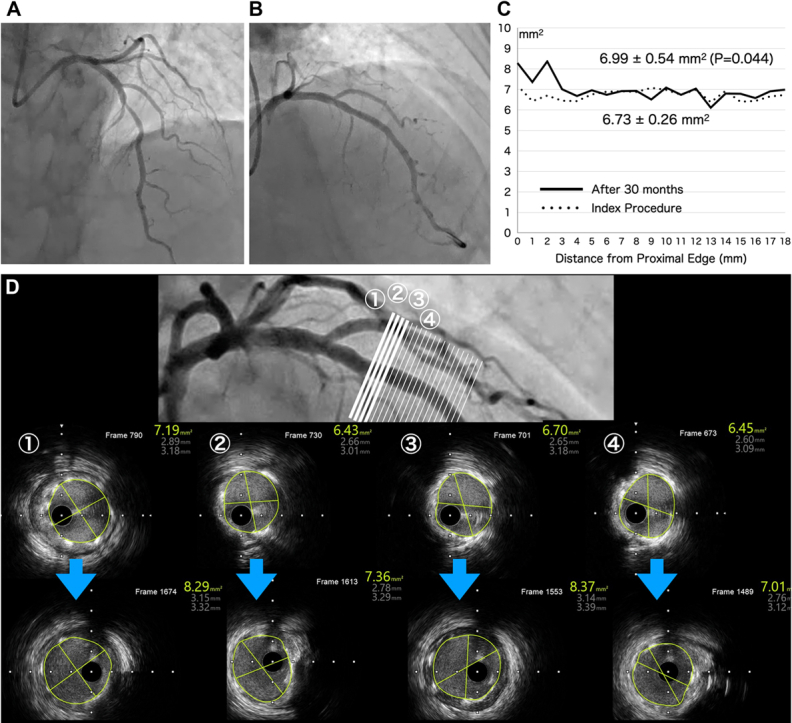

Substantial evidence has established the efficacy of drug-eluting stents (DESs) in coronary interventions, including the prevention of acute stent thrombosis and in-stent restenosis.^1^ However, the increasing rate of major adverse cardiovascular events (MACEs) over time without reaching a plateau has been observed, with rates as high as 20% within 5 years.^2^

**Take-Home Messages**

• The DynamX Bioadaptor preserves the hemodynamic modulation of vessels, thereby promoting positive remodeling inside the scaffold both with and without dominant endothelial proliferation for up to 30 months after PCI.
• Therefore, the Bioadaptor is a promising alternative to contemporary DESs.

Once implanted, coronary stents, including DESs, immobilize the vessel and restrict its natural movement and function, such as expansion, contraction, rotation, and vasomotion. This “caging” effect hinders proper hemodynamic modulation and can lead to complications, such as mechanical distortion, stent fracture, and subsequent stent thrombosis.^3^ Additionally, the restriction of natural vessel dynamics inhibits vasomotion and positive remodeling of the coronary artery, thereby contributing to a worsening prognosis by increasing the risk of MACEs, as mentioned earlier.^4^^,^^5^

The DynamX Bioadaptor (Elixir Medical Corporation) is an innovative cobalt-chromium platform with 3 helical strands connected by a bioabsorbable polymer. Similar to a conventional DES, these 3 interconnected strands initially provide immediate radial force against the vessel wall on delivery and dilation. After 6 months, the connecting polymer is resolved, and the device links are released, which then uncage the vessel.^6^ This design allows the Bioadaptor to offer adaptive, dynamic support to the diseased vessel, thus ensuring the restoration of hemodynamic modulation of the artery, including cyclic pulsatility, vessel compliance, and coronary blood volume.^7^ In addition, this unique mechanism contributes to long-term physiological remodeling of targeted lesions.^8^ Thus far, only limited data on vessel remodeling within the first 12 months following Bioadaptor implantation have been reported,^8^^,^^9^ and no data are available for periods beyond 1 year. Here we present the first 2 cases of physiological remodeling detected through intravascular imaging 30 and 36 months post-implantation of the Bioadaptor.

This study was conducted in accordance with the tenets of the Declaration of Helsinki, and written informed consent was obtained from all patients. The study protocol was approved by the Sakurakai Takahashi Hospital Institutional Review Board for the potential publication of this report. The data that support the findings of this case report are not publicly available because of privacy or ethical restrictions. However, deidentified data may be available from the corresponding author on reasonable request.

## Case Reports

### Case 1

**Figure 1:**
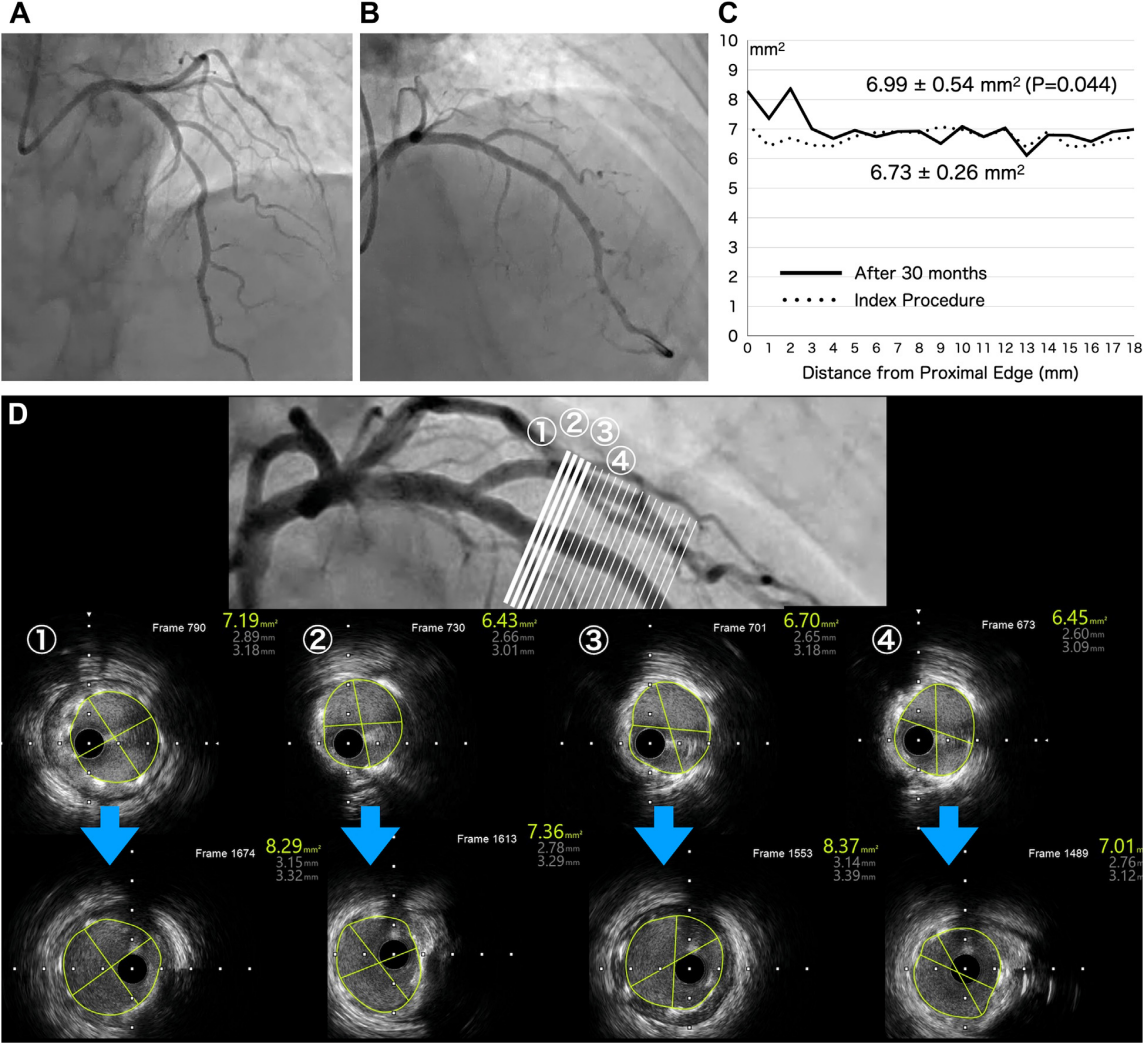
Imaging of the Left Anterior Descending Coronary Artery (A) Initial coronary angiography: Diagnostic coronary angiography shows significant stenosis in the mid-left anterior descending artery. (B) Final coronary angiography: Final angiography after percutaneous coronary intervention shows excellent dilatation of the stenotic lesion using the DynamX Bioadaptor (Elixir Medical Corporation). (C) Cross-sectional area of scaffold: The mean cross-sectional area of the scaffold at every 1 mm over its entire length after 30 months is significantly larger than that immediately after percutaneous coronary intervention (6.99 ± 0.54 mm^2^ vs 6.73 ± 0.26 mm^2^; *P* = 0.044). (D) Intravascular ultrasound findings: Findings at the proximal segment after the index procedure and those at 30 months indicate that the device lumen has become larger and the amount of atherosclerotic plaque outside the scaffold has reduced, compared with the initial amount.

A 59-year-old man with dyslipidemia and hypertension was admitted because of worsening exertional dyspnea. Coronary angiography (CAG) revealed significant stenosis of the mid-left anterior descending (LAD) artery (Figure 1A). Elective percutaneous coronary intervention (PCI) was subsequently performed for the LAD. A 6-F extra-backup guiding catheter was inserted through the distal right radial access. After crossing the lesion with a guidewire (SION blue wire, Asahi Intecc Co), intravascular ultrasound (IVUS) was conducted, revealing a lipid-rich plaque with minimal calcification in the lesion. Predilatation was carried out using a semicompliant balloon catheter (Traveler, 2.75 /15 mm, Abbott Vascular) at 14 atm. A 3.0/18-mm Bioadaptor was then successfully deployed at 10 atm. Postdilatation was performed using a noncompliant (NC) balloon catheter (NC Traveler, 3.0 /8 mm, Abbott Vascular) at 18 atm. The final CAG showed excellent dilatation of the stenotic lesion (Figure 1B). IVUS showed good scaffold expansion with sufficient cross-sectional area, even in regions with eccentric atherosclerotic distribution, and no evidence of device malapposition. At the 30-month follow-up, IVUS imaging revealed uniform scaffold expansion without significant neointimal proliferation in the scaffolded segment. The mean cross-sectional area of the scaffold at each 1-mm interval along its entire length was significantly larger than that immediately after PCI (6.99 ± 0.54 mm^2^ vs 6.73 ± 0.26 mm^2^; *P* = 0.044) (Figure 1C). In the proximal segment, the amount of atherosclerotic plaque outside the scaffold had decreased compared with the initial IVUS findings (Figure 1D).

### Case 2

**Figure 2:**
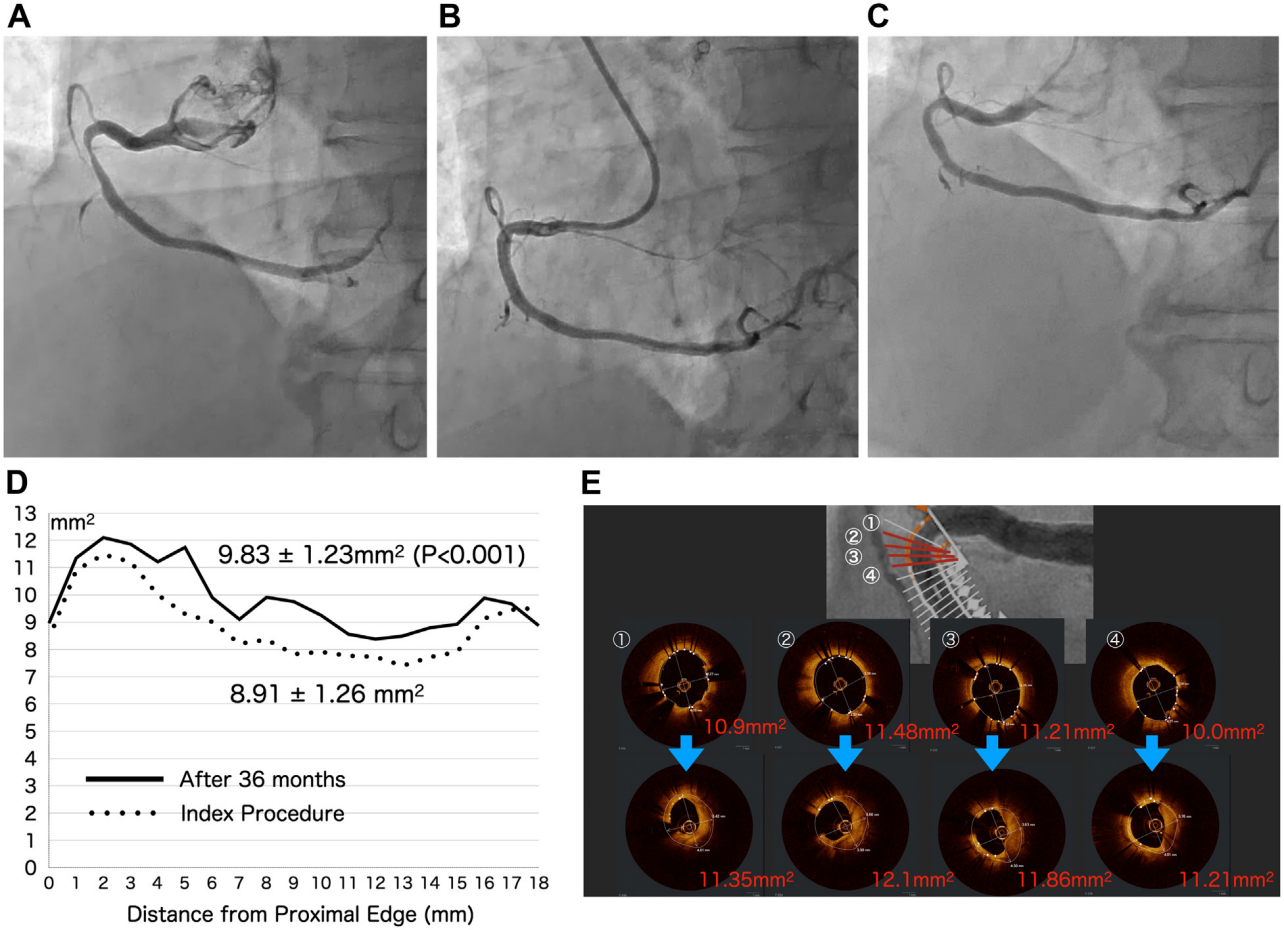
Imaging of the Right Coronary Artery (A) Initial coronary angiography: Diagnostic coronary angiography shows significant stenosis in the proximal right coronary artery. (B) Final coronary angiography: Final angiography performed after the percutaneous coronary intervention demonstrates excellent dilatation of the stenotic lesion using the scaffold. (C) Follow-up coronary angiography: The 36-month follow-up angiography indicates narrowing in the proximal part of the scaffold. (D) Cross-sectional area of scaffold: The mean device cross-sectional area is 8.91 ± 1.26 mm^2^ immediately after percutaneous coronary intervention and 9.81 ± 1.23 mm^2^ after 36 months (*P* < 0.001). (E) Optical coherence tomography image in stenosis: At the 36-month follow-up, the cross-sectional area of the scaffold over the lesion with neointimal hyperplasia, with a length of 5 mm has increased compared with the initial value.

A 58-year-old man with hypertension was admitted with exertional dyspnea. CAG revealed significant stenosis of the proximal right coronary artery (RCA) (Figure 2A). PCI was subsequently performed for the RCA. A 6-F SAL 1.5 guiding catheter (TAIGA, Medtronic) was inserted through the distal right radial access. After crossing the stenosis with a guidewire (SION blue wire), optical coherence tomography (OCT) was conducted, showing a noncalcified lipid-rich plaque with fibrous tissue in the lesion. Predilatation was performed with a semicompliant balloon catheter (Traveler, 3.0 /15 mm, Abbott Vascular) at 16 atm. A 3.5/18 mm Bioadaptor was then deployed successfully at 10 atm. Post-dilatation was subsequently performed using a noncompliant balloon catheter (NC Traveler, 3.5/8 mm) at 16 atm. The final angiogram revealed excellent dilatation of the stenotic lesion (Figure 2B) and the final optical coherence tomography (OCT) images showed optimal device expansion; the scaffold was expanded into a round shape, and no malapposition of the scaffold was observed. At the 36-month follow-up, coronary angiography indicated narrowing in the proximal part of the scaffold (Figure 2C), measuring 5 mm in length. OCT images showed that the scaffold maintained its round shape and expansion, although dominant endothelial proliferation was observed in the proximal scaffolded segment. The mean cross-sectional area at each 1-mm interval along its entire length of the scaffold was 8.91 ± 1.26 mm^2^ immediately after index procedure and 9.81 ± 1.23 mm^2^ at the 36-month follow-up (*P* < 0.001) (Figure 2D), with a high intimal tissue-strut coverage ratio of 98.7% (313 of 317 struts). Additionally, the mean cross-sectional area of the scaffold at the site of dominant endothelial proliferation, measuring 5 mm in length, was significantly larger at the 36-month follow-up than that initially measured (11.63 ± 0.41 mm^2^ vs 10.89 ± 0.64 mm^2^; *P* = 0.02) (Figure 2E).

## Discussion

To date, only a few studies have evaluated changes in the lumen size of vessels treated with the Bioadaptor scaffold by using intravascular imaging devices. In the first randomized trial comparing the Bioadaptor with the zotarolimus-eluting stent, the Bioadaptor demonstrated significant restoration of vessel compliance and cyclic pulsatility, as observed in IVUS and OCT examinations. Specifically, the in-device lumen area change during cardiac cycle was 7.5% for the Bioadaptor compared with 2.7% for the DES (*P* < 0.001) at the 12-month IVUS follow-up.^9^ Another study confirmed significant increases in both device area and vessel area, by 5% and 3%, respectively, 9 to 12 months after the index procedure.^8^ Furthermore, a preclinical study using Yucatan pigs showed late enlargement of the mean device area, from 7.02 ± 1.31 mm^2^ at post-PCI to 8.46 ± 1.31 mm^2^ at 24 months.^4^ These results provide evidence of the unlocking of the Bioadaptor scaffold and the subsequent release of the coronary artery. Thus, lumen enlargement of the device in response to positive vessel remodeling, which may be associated with neointimal hyperplasia or neoatherosclerosis, could be expected even in the long term. However, no studies have yet evaluated the change in lumen size of the device beyond 12 months in humans.

In the present study, physiological remodeling detected 30 or 36 months after Bioadaptor implantation revealed the following: 1) the mean cross-sectional area measured across the entire device length was significantly larger than that measured during the index procedure in both cases; 2) regression of lipid plaque outside the scaffold, accompanied by enlargement of the device area, was observed in the plaque-rich lesion in Case 1; and 3) the mean cross-sectional area of the scaffold at the site of narrowing, as a result of dominant endothelial proliferation, was significantly larger at the 36-month follow-up compared with the initially measured area in Case 2.

Specifically, the mean device area increased by 3.9% (*P* = 0.044) and 10.3% (*P* < 0.001) in Cases 1 and 2, respectively. Furthermore, in Case1, the regression of the lipid plaque and symmetrical expansion of the scaffold were observed on IVUS examination, where scaffold had previously expanded asymmetrically because of accumulation of lipid-rich plaque at the index procedure (from 7.19 mm^2^ to 8.29 mm^2^). These findings can be attributed to aggressive lipid-lowering therapy. However, imaging modalities other than IVUS, such as OCT and/or virtual histology, could provide a better understanding of the mechanisms behind plaque regression or potential late-acquired malapposition. In Case 2, no clinically relevant lumen narrowing at the proximal site of the scaffold was observed. In this lesion, although the inner lumen of the vessel became smaller, the device lumen enlarged by 6.7%. This finding may suggest that significant positive remodeling had occurred in response to the progression of intimal hyperplasia within the scaffold, a phenomenon similar to what is commonly observed during the progression of atherosclerosis in native coronary arteries. These phenomena may also indicate that mere uncaging of the vessel will not resolve the issue of in-stent restenosis.

These 2 cases highlight the unique features of the Bioadaptor, particularly its ability to preserve hemodynamic modulation of vessels and promote positive remodeling, regardless of the progression of intimal hyperplasia after PCI.

## Conclusions

The Bioadaptor scaffold showed significant enlargement 30 and 36 months after PCI. The enlargement was similarly observed in lesions with significant neointimal hyperplasia in the scaffold. Thus, the Bioadaptor is considered a promising alternative to contemporary DESs. Further studies with larger cohorts are warranted.

## Funding Support and Author Disclosures

The authors have reported that they have no relationships relevant to the contents of this paper to disclose.
